# Synthesis of Silver Nanoparticles Using Aqueous Leaf Extract of *Mimosa albida* (Mimosoideae): Characterization and Antioxidant Activity

**DOI:** 10.3390/ma13030503

**Published:** 2020-01-21

**Authors:** Fernanda Pilaquinga, Dennis Amaguaña, Jeroni Morey, Mauricio Moncada-Basualto, Josué Pozo-Martínez, Claudio Olea-Azar, Lenys Fernández, Patricio Espinoza-Montero, Eliza Jara-Negrete, Lorena Meneses, Fernanda López, Alexis Debut, Nieves Piña

**Affiliations:** 1 School of Chemical Sciences, Pontificia Universidad Católica del Ecuador, Av. 12 de octubre 1076, Apartado 17-01-2184, Ecuador; d-nena1996@hotmail.com (D.A.); lmfernandez@puce.edu.ec (L.F.); pespinoza646@puce.edu.ec (P.E.-M.); enjara@puce.edu.ec (E.J.-N.); lmmeneses@puce.edu.ec (L.M.); 2 Department of Chemistry, University of the Balearic Islands, Cra. de Valldemossa Km. 7.5, 07122 Palma de Mallorca, Spain; jeroni.morey@uib.es; 3 Department of Inorganic and Analytical Chemistry, University of Chile, Sergio Livingstone 1007, Independencia, Santiago 233, Chile; m.moncada.ba@gmail.com (M.M.-B.); jspozom@yahoo.es (J.P.-M.); colea@uchile.cl (C.O.-A.); 4 Departamento de Química, Universidad Simón Bolívar, Apartado, Caracas 89000, Venezuela; 5 School of Agricultural & Environmental Sciences, Pontificia Universidad Católica del Ecuador Sede Ibarra, Jorge Guzmán Rueda Ave., Ibarra 100150, Ecuador; mflopez2@pucesi.edu.ec; 6 Centro de Nanociencia y Nanotecnología, Universidad de las Fuerzas Armadas ESPE, Sangolqui 170501, Ecuador; apdebut@espe.edu.ec

**Keywords:** silver nanoparticles, *Mimosa albida*, polyphenolic total content, antioxidant activity

## Abstract

The search for sensitive and rapid analytical techniques for the determination of natural antioxidants is an area in constant growth due, among other aspects, to the complexity of plant matrices. In this study, silver nanoparticles prepared with the aqueous extract of *Mimosa albida* leaves were used to assess their polyphenolic content and antioxidant capacity. Silver nanoparticles were characterized by different techniques. As a result, nanoparticles of 6.5 ± 3.1 nm were obtained. The total phenolic content in the extract was 1320.4 ± 17.6 mg of gallic acid equivalents GAE· 100 g^−1^ and in the nanoparticles 257.3 ± 5.1 mg GAE· 100 g^−1^. From the phenolic profile analyzed by ultra high-performance liquid chromatography (UPLC) with a diode-array detector (DAD), the presence of apigenin and luteolin in the plant extract is postulated. The antioxidant capacity measured by oxygen radical absorbance capacity ORAC-fluorescein assay was 86917 ± 6287 and 7563 ± 967 µmol ET g^−1^ in the extract and nanoparticles respectively. Electrochemical analysis by cyclic voltammetry (CV) confirmed the effective reduction capacity of the *Mimosa albida* leaves extract to reduce Ag ions to AgNPs and differential pulse voltammetry (DPV) suggested the presence of two main reducing agents in the extract. From this study, it was concluded that the aqueous extract of *Mimosa albida* contains reducing agents capable of synthesizing silver nanoparticles, which can be used in the phytochemical industry.

## 1. Introduction

The study of silver nanoparticles (AgNPs), has undergone a breakthrough in recent decades, mainly due to their optical [1,2] and antimicrobial properties [3,4,5,6]. AgNPs can be prepared by means of physical, chemical, electrochemical and biological methods [7,8]. In the chemical reduction method, a metal salt is used as a precursor and a reducing agent [9]. Among the chemical reducers, those of greatest interest are those of natural origin [10,11,12,13], whereby stable colloidal dispersions are obtained. This method represents a very interesting alternative, since it avoids the use of toxic chemical reducers [14]. It also offers possibility of controlling the size of the nanoparticles by varying the concentration of the plant extract without using surfactants [14] or stabilizers [15], which is very advantageous from an economic and environmental point of view [16]. Compared to biological methods that use fungi and bacteria, synthesis with plant extracts is much easier and faster [17,18]. The parameters to be controlled in the synthesis of AgNPs with plant extracts are: the concentration of the metal precursor and extract, temperature, pH and contact time [19]. The use of plant extracts as reducing agents has several advantages such as: Accessibility and safety when handling. In addition, the presence of secondary metabolites in the extracts [20] promote the reduction of ions from Ag^+^ to Ag^0^ [10].

Among the most studied metabolites, there are polyphenolic compounds, glycosides with reducing terminal ends and organic acids with antioxidant capacity [15,18]. Phenolic compounds constitute one of the most numerous and representative groups of secondary plant metabolites [21]. Its importance lies in its participation in physiological processes, cellular metabolism, growth, reproduction, germination processes, as well as defense against pests and predators, among others [21,22,23]. Recent studies [24,25], have demonstrated their significant antioxidant activity [21], which may be beneficial to human health [26]. The antioxidant capacity of polyphenols, produced by cellular metabolism, is due to the capture and inactivation of free radicals and reactive oxygen species (ROS) [27]. One mechanism of antioxidant action of polyphenols involves the transfer of hydrogen atoms and electrons thus generating more stable radicals [28].

The development of efficient procedures for extraction, proper analysis and characterization of phenolic compounds from different sources is a difficult task due to structural diversity of compounds, complexity of natural sources and their interaction with other components in the matrix [29]. In recent years, different analytical approaches have been tried to assess the total polyphenol content and antioxidant capacity in biological and food samples [23,30]. Special attention has been given to tests that have the advantage of sensitivity, speed and simplicity. The use of AgNPs was introduced as a novel tool for evaluation of total polyphenolic content and antioxidant capacity [31]. The optical detection of AgNPs is produced by surface plasmon resonance (SPR), which refers to the collective oscillation of the conduction electrons of the metal. The absorption band corresponding to nanoparticles varies according to the type of plant extract used and size obtained [32]. Several studies have been described regarding the determination of phenolic compounds and antioxidant capacity in AgNPs synthesized with extracts of different herbaceous species [33,34].

**Figure 1:**
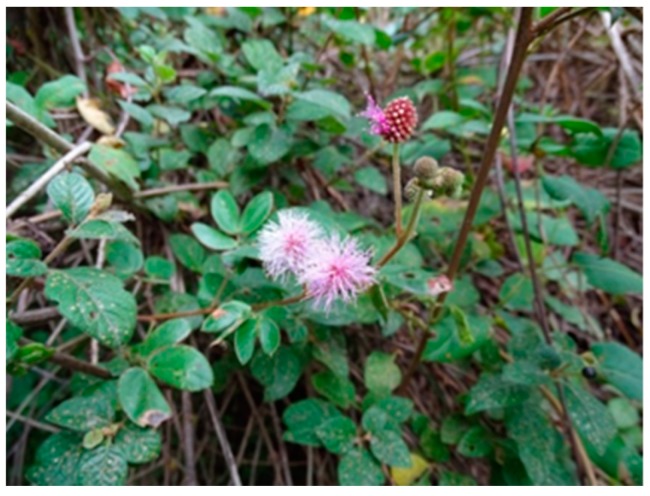
*Mimosa albida* plant.

*Mimosa albida* (Figure 1) is a plant native to Ecuador. It is known as uña de gato (cat’s claw), mimosa, sarza, tapa vergüenza (cover shame) or dormilona (sleepyhead) [35,36]. It is traditionally used in the treatment of heart disease, migraine, insomnia, fever, cough, rheumatism, liver and kidney pain, and as a healing agent [37]. The phenolic compounds present confer analgesic, healing and antibacterial properties [38]. Studies of this plant are not extensive, however its use as a contraceptive is suggested [39]. In similar species such as *M. púdica* antiparasitic activity is described [40,41], as a larvicide [42] and its use in electrochemical detection of AgNPs [40]. Also noted the antibacterial and antifungal action of *M. tenuiflora* [43].

In this study, AgNPs synthesized with the aqueous extract of *M. albida* (*Ma*) leaves were used as an analysis tool in the determination of polyphenolic content and antioxidant capacity of extract, as a potential source of important polyphenolic compounds in the food or pharmaceutical industries.

## 2. Materials and Methods

### 2.1. Preparation of *Mimosa Albida* Leaf Extract

Leaves of *M. albida* were collected in the Loma de Guayabillas, located in the city of Ibarra-Ecuador at an altitude of 2200–2475 m.a.s.l. (geographical coordinates: 00°20′22″ N; 78°00′25″ O X: 822003.13; Y: 374482.29). Leaves were disinfected with 60% ethanol, pressed and kept in quarantine in a cold room at −10 °C for 48 h. Subsequently, they were dried at 60 °C in a dehydrator (Termokool, Barcelona, Spain) for 20 min. Finally, the sample was pulverized in a crusher (Retschgm 200, Haan, Germany) at 4500 rpm for 3 min. According to the literature, different conditions were tested in terms of quantity of plant sample (0.25 to 2 g), temperature (20 to 70 °C) and contact time (25 to 60 min) [44]. For the optimized method, 1 g of sample was mixed in 20 mL of distilled water, temperature controlled at 60 °C, constant stirring at 4500 rpm for 30 min using a heating plate (Velp, Usmate Velate, Italy). Subsequently, the sample was centrifuged at 8000 rpm in a centrifuge (K PLC series, San Borja, Peru), filtered using Whatman No. 4 paper, and stored at 4 °C.

### 2.2. Synthesis of *M. Albida* Silver Nanoparticles (MaAgNPs)

For the synthesis of *Ma*AgNPs using the aqueous extract of *M. albida*, the following parameters were varied: concentration of silver nitrate (0.5 to 5 mM), volume of extract (1 to 3 mL), pH (7 to 11), temperature (20 to 60 °C) and contact time (10 to 50 min) [45]. For the conditioned method, 20 mL of a 1 mM silver nitrate solution (Merck 99.9%, Darmstadt, Germany), 2 mL of the aqueous extract at pH 8 were taken at a temperature of 60 °C with constant stirring at 4500 rpm for 50 min. The colloidal solution was stored in amber glass jars at 4 °C.

### 2.3. Characterization of MaAgNPs

For the ultraviolet–visible (UV–Vis) characterization, double beam spectrophotometer Cary 60 (Agilent Technologies, Santa Clara, CA, USA) and the Cary WinUV 3.1 software was used. The wavelength range used was 350 to 800 nm with a resolution of 1 nm. Colloidal solutions were diluted in 1:10 portion with distilled water. Transmission Electron Microscopy TEM (FEI Tecnai G2 Spirit Twin, Hillsboro, OR, USA) analyses were realized at 80 kV. 138 nanoparticles were observed, and the average size has been determined using the Fiji-software [46]. Samples were prepared on formvar carbon coated 300 mesh copper grids. A field-emission gun scanning electron microscope (FEG-SEM Tescan Mira 3, Brno, Czech Republic), 1.2 nm resolution at 30 keV, was used to determine elemental composition by energy-dispersive X-ray spectroscopy (EDX) using a Bruker X-Flash 6|30 detector (Bruker, Berlin, Germany), with a 123 eV resolution at Mn Kα. Samples have been fixed in a stub previously prepared with two layers of double coated carbon conductive tape and samples were then covered with a 20 nm conductive gold layer (99.99% purity) using a sputtering evaporator Quorum Q150R ES (Quorum Technologies, East Sussex, UK). Dynamic light-scattering (DLS) measurements for determining the average size of the silver nanoparticles were carried out using the LB-550 analyzer (HORIBA, Kyoto, Japan). One drop of 200 µL of a filtered sample, 220 nm Whatman filter, was mixed into 3 mL ethanol. The data analysis was performed with HORIBA LA-920W, integrated software Version 3.57. XRD spectra was obtained with a (PANalytical EMPYREAN, Almelo, The Netherlands) diffractometer within a 2θ configuration (generator-detector) equipped with a Cu K-alpha (λ = 1.541 Å), a nickel filter and an X’CELERATOR detector. Liquid sample preparation was dried at 30 °C on a microscope slide to obtain a thin layer and avoid any organic degradation. Following this, six XRD diffraction patterns were acquired from 5° to 90° (θ–2θ configuration).

### 2.4. Polyphenolic Total Content

The determination of total polyphenols was carried out using the Folin–Ciocalteu spectrophotometric method [47]. To 50 µL of extract and AgNPs, 50 µL of Folin-Ciocalteu reagent (Merck 99.99%, Darmstadt, Germany) were added. The mixture was homogenised and allowed to incubate for 5 min. Then 150 µL de Na_2_CO_3_ al 20% (m/v) and 700 µL of distilled water were added and the reaction mixture was homogenized and incubated for 30 min. Subsequently, the absorbance at 760 nm in a micro-plate reader by the detection of multiple Synergy HT, from (Bio-Tek Instruments, Winooski, VT, USA) was recorded. The results are expressed in gallic acid equivalents (mg GAE· 100 g^−1^ of dry mass). A calibration curve with a concentration of gallic acid between 5–55 µg Ml^−1^ was used.

### 2.5. Phenolic Profile

The phenolic profile was obtained by ultra high-performance liquid chromatography (UPLC Waters Acquity, Milford, CT, USA) with an Atlantis C18 column (4.6 × 150 mm; 5 μm) and diode array detector (PDA 190 at 800 nm, resolution 1.2 nm). The mobile phase water (A)-acetonitrile (B), with 1% formic acid as a modifier, was applied at a flow of 0.5 mL/min and with gradient programming: 85% B–5 min, 80% B up to 7 min, 75% B up to 10 min, 65% B up to 12 min, 50% B up to 15 min, 85% B up to 30 min. Column temperature was kept constant at 25 °C and 25 µL was used as the injection volume in all samples. The standards for identification were: gallic acid, chlorogenic acid and *p*-cumaric acid.

### 2.6. Antioxidant Activity by Oxygen radical absorbance capacity-Fluorescein assay ORAC-FL 

The ORAC-FL analyses were carried out on a plate reader (EnSpire multimode Perkin-Elmer, Waltham, MA, USA) of 96 wells of white polystyrene. The fluorescence was read from the top, with an excitation wavelength of 485 nm and an emission of 528 nm. The reaction was carried out in 75 mM (pH 7.4) phosphate buffer. 150 µL of fluorescein solution (FL) (40 nM, final concentration) and 25 µL of the extract and nanoparticles were added to each well of the plate. The sample was incubated for 7 min at 37 °C and then 25 µL of a 2,2′-azo-bis(2-methylpropionamidine) dihydrochloride (APPH) solution (Sigma-Aldrich 97%, St. Louis, MO, USA) 18 mM final concentration, were added. Fluorescence was recorded every minute for 120 min and it was used as FL and AAPH control, using solvent instead of the solution of the extracts [48,49,50]. A Trolox (3 μM to 20 μM) calibration curve was prepared as a reference antioxidant. The inhibition capacity was expressed as μmol Trolox equivalent per gram of extract. All reaction mixtures were prepared in triplicate and at least three independent assays were performed for each sample. The area under the fluorescence decay curve (ABC) was calculated by integrating the fluorescence decrease.

### 2.7. Antioxidant Activity by Voltamperommetric Analysis

To study the electrochemical characteristics of the modified electrodes a CH-Instruments Potentiostat (Tennison Hill Drive Austin, Bee Cave, TX, USA), Model 700D, was used coupled to a conventional 15 mL three-electrode reaction cell. Glassy carbon (GC) was used as working electrodes, an Ag/AgCl electrode was used as reference, and a platinum wire (0.5 cm diameter and 3 cm long) was the counter-electrode. The cyclic voltammetry and differential pulse voltammetry were conducted in a background electrolyte solution of 0.1 mol L^−1^ sodium acetate, in a range from −0.5 to 1.3 V.

### 2.8. Data Analysis

For preparation of *M. albida* extract, three factors at five levels were tested and *Ma*AgNPs had five factors tested at four levels respectively. Total phenolic content, and antioxidant activity by ORAC-FL and cyclic voltammetric, were performed in five repetitions (N = 5). Normal distribution of data was verified by Student’s *t*-test with a significance of 95% (*p* < 0.05). The obtained results were expressed as mean ± standard deviation. Data processing was performed with the Origin Pro 8.5 SR2 program (Originlab Corporation, Northampton, MA, USA).

## 3. Results

### 3.1. Synthesis and Characterization of MaAgNPs

**Figure 2:**
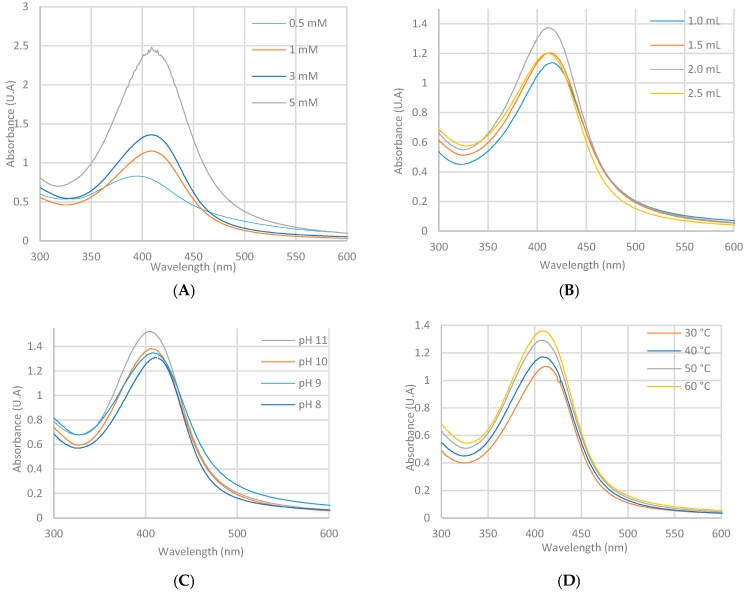

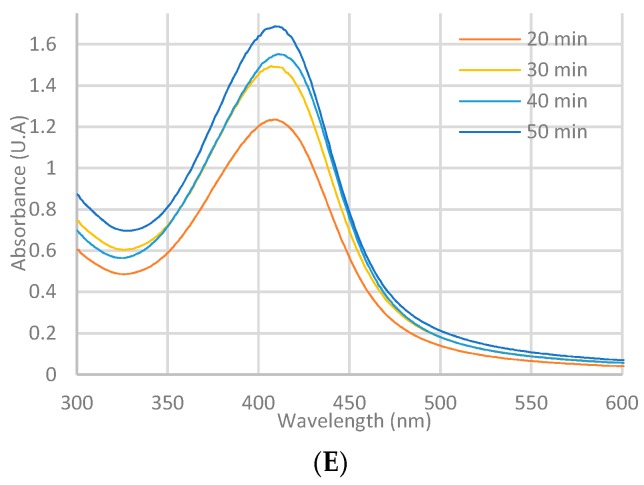
Ultraviolet–visible (UV–Vis) spectra of synthesis parameters of *M. Albida* silver nanoparticles (*Ma*AgNPs) concentration of silver nitrate (**A**), volume of extract (**B**), pH (**C**), temperature (**D**) and contact time (**E**).

Optimization of the *Ma*AgNPs synthesis was monitored by UV–Vis spectrophotometry, as shown in Figure 2. The parameters of silver nitrate concentration (Figure 2A), extract volume (Figure 2B), pH (Figure 2C), temperature (Figure 2D) and contact time (Figure 2E) were varied. To study the behavior of the nanoparticles against the concentration of the precursor salt of AgNO_3_, concentrations of 0.5, 1, 3 and 5 mM were used.

**Figure 3:**
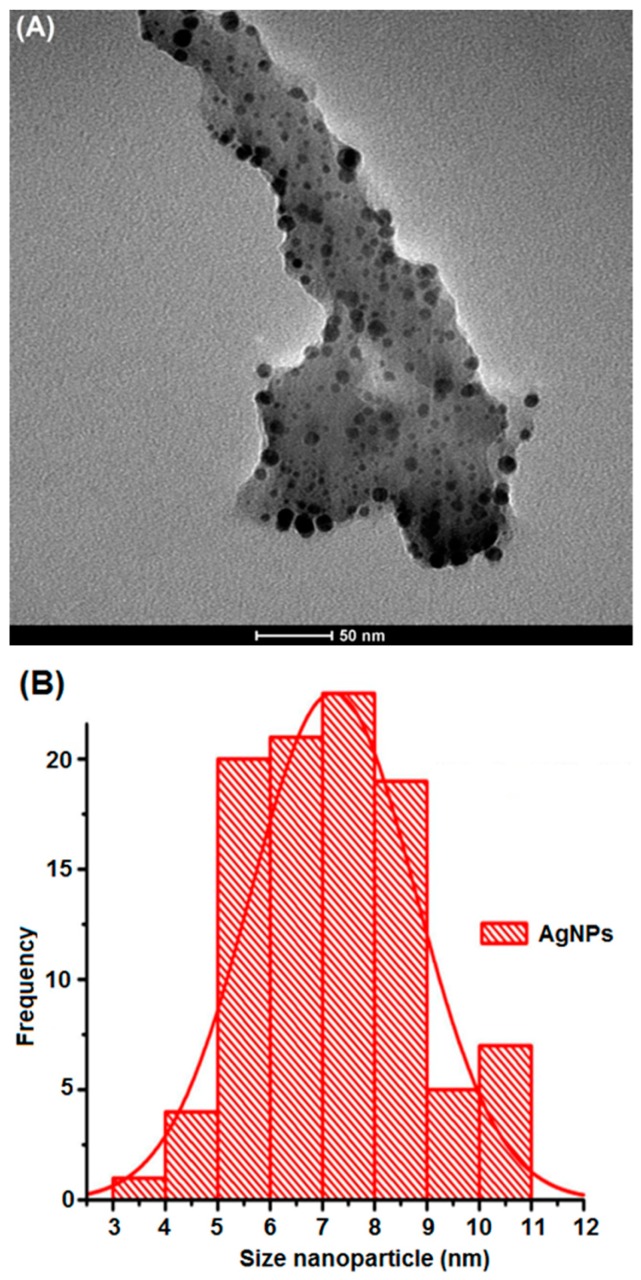
*Ma*AgNPs transmission electron microscopy (TEM) micrograph taken at 80 kV (**A**) and frequency histogram (**B**).

Average size value measured by transmission electron microscopy (TEM) was found to be 6.5 nm ± 3.1 nm (Figure 3A). The TEM micrograph (Figure 3A) and the normalized frequency histogram of the size values of *Ma*AgNPs (Figure 3B) are shown.

**Figure 4:**
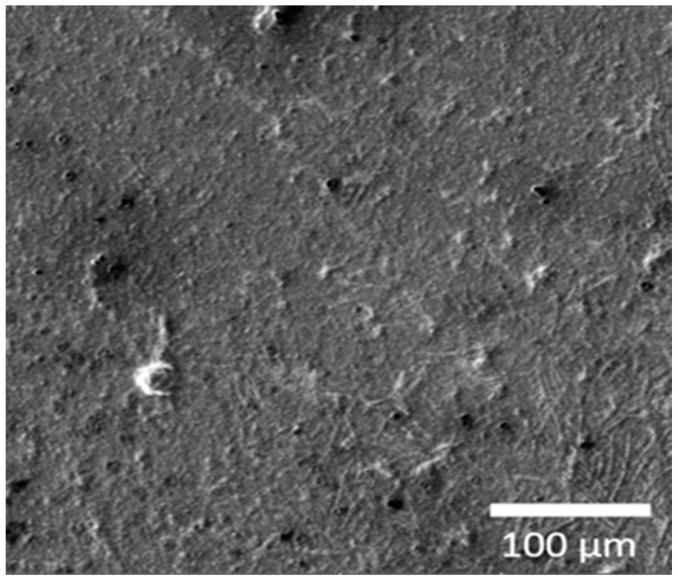
Field-emission gun scanning electron microscope (FEG-SEM) micrograph of *Ma*AgNPs taken at 5 kV.

The FEG-SEM micrograph of *Ma*AgNPs is shown in Figure 4. The normalized weight % average values of each element and the standard deviation (σ) are listed in Table 1.

**Figure 5:**
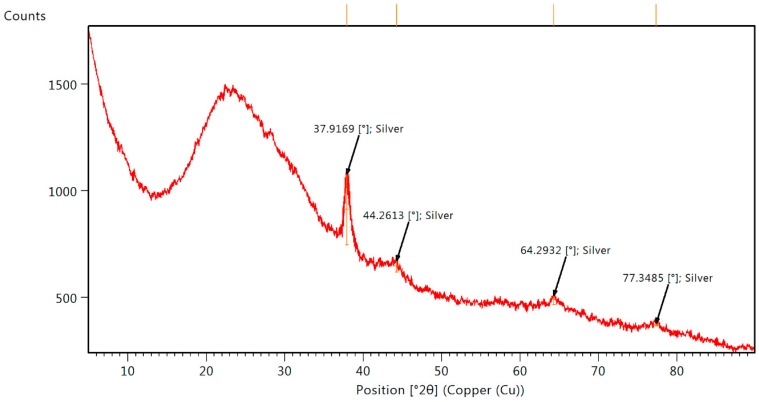
X-ray diffraction (XRD) pattern of *Ma*AgNPs.

The *Ma*AgNPs crystalline nature was confirmed from the XRD patterns (Figure 5).

### 3.2. Polyphenolic Total Content

Table 2 shows the total polyphenol content found in *M. albida* extract and *Ma*AgNPs.

### 3.3. Phenolic Profile

**Figure 6:**
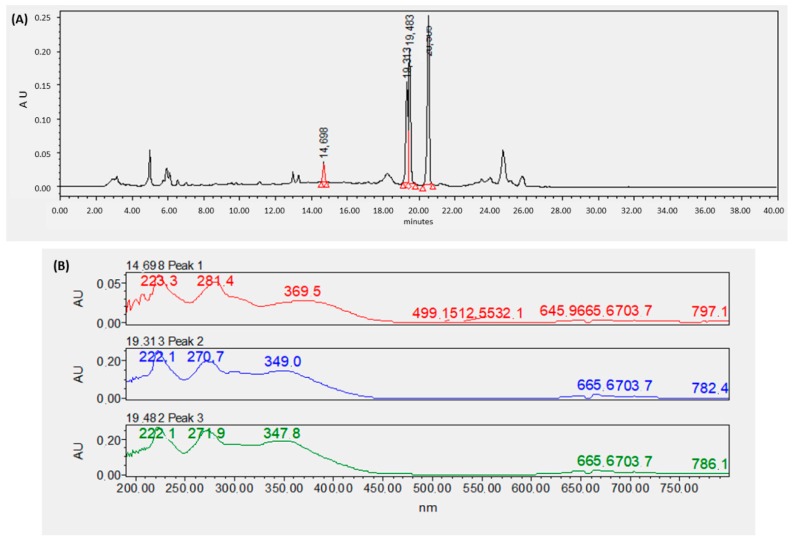

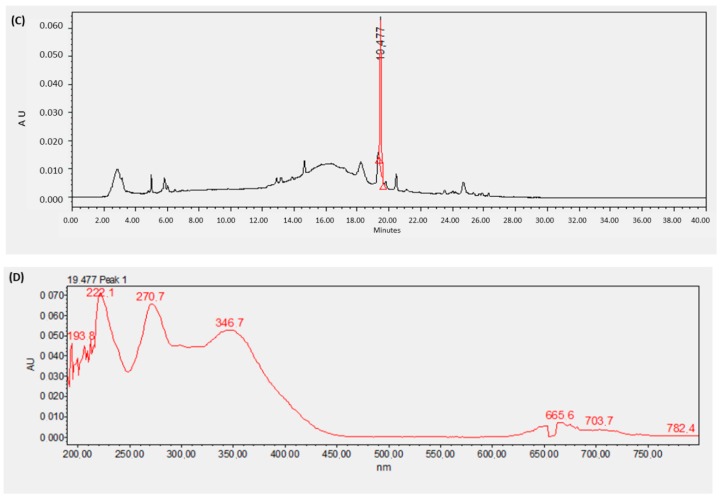
Ultra high-performance liquid chromatography (UPLC) chromatograms of *M. albida* extract (**A**) and *Ma*AgNPs synthesized with it (**C**). UV-Vis spectra are shown for the main components of the extract (**B**) and *Ma*AgNPs (**D**).

Figure 6 shows ultra high-performance liquid chromatography with diode-array detector (UPLC-DAD) chromatograms (Figure 6A,C) and UV-Vis spectra of *M. albida* extract and *Ma*AgNPs (Figure 6B,D) respectively.

### 3.4. Antioxidant Activity by ORAC-FL

Table 3 shows the results of the antioxidant capacity measurement by ORAC-FL in *M. albida* extract and *Ma*AgNPs, expressed as μmol Trolox equivalent.

### 3.5. Antioxidant Activity by Voltammetric Analysis

**Figure 7:**
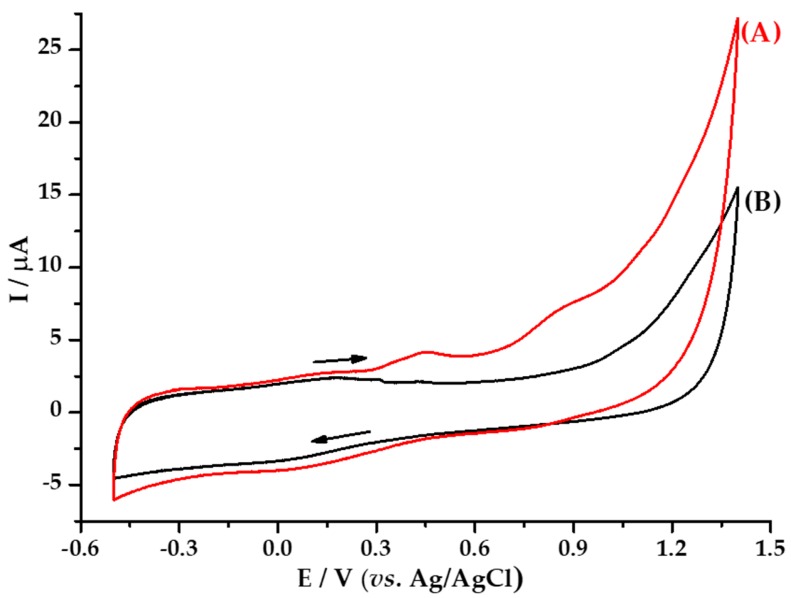
Cyclic voltammograms of: (**A**) *M. albida* extract in 0.10 mol L^−1^ sodium acetate solution, (**B**) blank acetate solution; on glassy carbon (GC) work electrode. Scanning rate 50 mV/s vs. Ag/AgCl at 25 °C.

**Figure 8:**
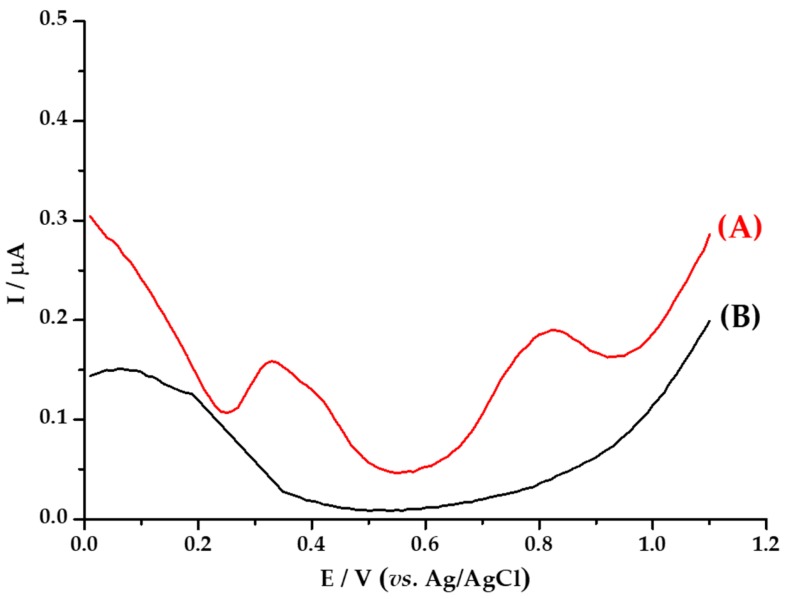
Differential pulse voltammetry analysis of: (**A**) *M. albida* extract electrode in 0.10 mol L^−1^ sodium acetate solution, (**B**) blank acetate solution; on GC work electrode. Scanning rate 50 mV/s vs. Ag/AgCl at 25 °C.

**Figure 9:**
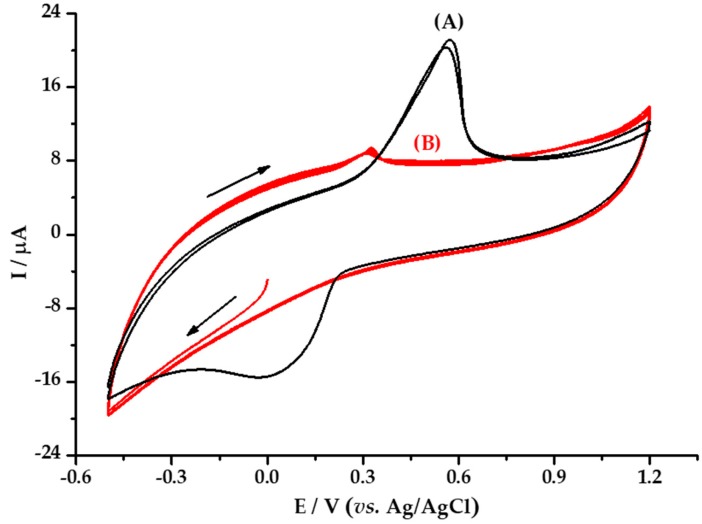
Cyclic voltammograms of: (**A**) 5 mmol L^−1^ AgNO_3_ + 0.10 mol L^−1^ sodium acetate solution, (**B**) *Ma*AgNPs in 0.10 mol L^−1^ sodium acetate solution; on GC work electrode. Scanning rate 50 mV/s vs. Ag/AgCl at 25 °C.

Three analysis were carried out by voltammetry. Figure 7 shows the cyclic voltamperogram of the *M. albida* extract; Figure 8 shows the voltamperogram of *M. albida* extract by differential pulse voltammetry (DPV); and Figure 9 shows the cyclic voltamperogram of *Ma*AgNPs.

## 4. Discussion

### 4.1. Synthesis and Characterization of MaAgNPs

To study the behavior of the nanoparticles against the concentration of the precursor salt of AgNO_3_, concentrations of 0.5, 1, 3 and 5 mM were used. The maximum absorption band was presented when using a 1 mM solution with a wavelength of 412 nm (Figure 2A). Depending on the variation in the volume of extract, 1 to 3 mL was used, and the maximum absorption band at 412 nm (Figure 2B) was shown with 2 mL. The pH varied in the range of 7 to 11, obtaining the maximum absorbance band at pH 8 at 411 nm (Figure 2C). The temperature change from 20 to 60 °C showed a maximum absorption band at 60 °C at 413 nm (Figure 2D). In relation to the reaction times, 10, 20, 30, 40 and 50 min were considered, showing the maximum absorption band at 50 min at 415 nm (2E). The UV–Vis spectrum with all the resulting variables showed a maximum at 411 nm, a value that was confirmed with the second derivative spectrum. In similar results using *Mimosa púdica* species, AgNPs presented a maximum absorption at 420 nm [51].Other studies have reported a maximum absorption at 420 nm and 430 nm; and sizes that vary from 25–60 nm [40,41].

The TEM image (Figure 3) shows *Ma*AgNPs with an average size of 6.5 ± 3.1 nm, clearly surrounded by an organic phase, presumably, the *M. albida* extract. The mean value obtained by DLS was 9.3 ± 0.3 nm. This value was, as expected, higher than the TEM result. This is mainly due to the organic coating of the silver nanoparticles, that can be estimated around 2.8 nm [52]. *M. púdica* reflects greater concordance with *M. albida*, where AgNPs are shown with an absorbance UV–VIS at 412 nm and an average size of 6 nm [53]. The standard deviation corresponds to the variability of sizes, since they are not uniforms.

The SEM image (Figure 4) shows the homogeneous distribution of silver nanoparticles as bright spots. In order to avoid biased determinations of the chemical compositions of the sample, we have averaged the energy dispersive X-ray (EDX) spectra obtained from 25 points grid on a total area of at least 6.40 mm^2^. The presence of each element is denoted by the normalized weight percentage (norm. wt.%), which is the percentage in weight, supposing that the chosen elements represent the total composition of the sample. C, O, Na, Mg, S, K and Ca detected (Table 1) are attributable to the organic extract. Silver is also detected, which confirms the nanoparticles formation.

In XRD spectra (Figure 5), the Bragg reflection peak at 37.92° coincides with the face centered cubic structure (FCC) of silver (ICSD: 604629). The (111) lattice parameter and highest intensity plane is well matched and agrees with other reported patterns [52,54]. Further peaks are observed around 44° (002), 64° (022) and 77° (113). Further peaks are observed around 44° (002), 64° (022) and 77° (113). They correspond to the same silver diffraction pattern, mixed with the *M. albida* extract, which indicates that the *Ma*AgNPs process is the unique inorganic compound that is created and that no silver nitride or silver nitrate compounds were created during the synthesis, as their mayor diffraction peak is not observed at 29.7° and 40.1° respectively [55,56]. The Debye Scherrer’s equation at highest reflection peak (full width at half maximum, FWHM = 0.643°) gives a 13.6nm approximated size value for the silver nanoparticles. This calculated value is in good agreement with the TEM measurements.

### 4.2. Polyphenolic Total Content

Phytochemical screening was analysed qualitatively in *M. albida* extract and *Ma*AgNPs previously by ferric chloride and the Shinoda test as a positive. Fehling’s test for reducing sugars was negative [51]. The total polyphenolic content carried out using the Folin–Ciocalteu method, which is based on the reduction of the Folin reagent, due to the oxidation of the phenolic compounds. In Table 2, the results obtained are shown, where it was found that in the *M. albida* plant the polyphenol content is high with respect to the total polyphenols anchored to the *Ma*AgNPs, due to some polyphenols forming a complex with the silver nanoparticle, preventing the reduction of Folin’s reagent. A recent study determined that plants with high reducing capacity are excellent sources for the synthesis of metal nanoparticles due to the presence of polyphenolic compounds with antioxidant capacity [57]. Another study indicated that polyphenols can stabilize and support AgNPs complex formation, due to their reducing power [46].

The difference observed in the total polyphenol content can also be attributed to the loss of reducing capacity of phenolic compounds present in the extract, which were used in the reduction of silver salt. The latter allows us to postulate that variation in total polyphenol content can account for the effective reducing capacity of the extract as an approach to enhance the use of this technique for the determination of the effective antioxidant capacity of naturally occurring compounds [34,44].

### 4.3. Phenolic Profile

The chromatogram of *M. albida* extract shows (Figure 6) two main peaks (Figure 6A tr 19.313 min and 19.482 min) that could not be identified with the available standards (gallic acid 3.21, chlorogenic acid 12.5 min, *p*-cumaric acid 10.24 min). These UV–Vis spectra (Figure 6B) show distinctive bands at 222, 270–271 and 348–349 nm, which is an indication of the presence of flavonoids, specifically flavones or flavonols, as all their structures have two absorption bands in the following ranges: 240–290 and 310–370 nm [58]. When analysing the nanoparticles obtained via the process of green synthesis, there is a coincidence with the crude extract, with the compound being quite similar in regards to both retention time (19.477 min, Figure 6C) and UV–Vis spectrum (Figure 6D; bands 222, 270 and 347 nm). Although this is the first study of the extract of *M. albida*, several sources describe the presence of flavones and flavonols in Mimosa species, for instance: Apigenin [59], luteolin [60], quercetin and their glycosides [61], thus allowing us to ratify the interpretation of the information obtained by chromatography. It is important to note that quercetin spectrum has bands at different wavelengths than those observed for *M. albida* extract, with the presence of a flavone being more likely. The polyphenolic compound is expected to be present in its glycosylated form, as this chemical form is more common in plant extracts, but also due to its higher solubility in water (extracting media). It is important to note that glycosides do not have a major effect on bands position within UV spectra [58].

### 4.4. Antioxidant Activity by ORAC-FL

Antioxidant capacity was determined by the ORAC method, which is based on the ability to trap oxygen-centered radicals. Table 3 shows the results of antioxidant capacity of aqueous extract and *Ma*AgNP, where it is observed that *Mimosa albida* extract has a great antioxidant capacity than *Ma*AgNP in µmol TE/g extract, determined through a Trolox calibration curve.

It was determined that antioxidant capacity the extract decreased when forming the *Ma*AgNPs, due to the ORAC method, as previously mentioned in the section on total polyphenolic content. Similar studies using *M. púdica* with AgNPs, have not reported their antioxidant activity [60]; only antioxidant activity in leaves, seeds and stems measured by DPPH method have been reported [62]. AgNPs formation is due to the reduction of silver ions by action of reducing functional groups such as hydroxyl, which is evidenced in the results of antioxidant capacity [53]. According to the literature, there are at least two types of mechanisms for deactivation of free radicals by hydrogen atom transfer (HAT) and electronic transfer (ET), and these reactions can occur in parallel, the dominant mechanism being determined by the structure and property of antioxidant compound [63]. In addition, the presence of reducing agents affects HAT mechanisms, which was evidenced in the variation of antioxidant capacity between the extract of *M. albida* and the *Ma*AgNPs. The decrease in the antioxidant capacity of *Ma*AgNPs, in relation to aqueous extract, is interesting from the point of view of the antioxidant capacity that is actually used in reduction of silver salt for the formation of nanoparticle. Therefore, the difference between the two capacities would account not only for the antioxidant capacity of the extract, but rather for the effective antioxidant capacity of the extract. From this methodology, it would be possible to analyze the effective antioxidant capacity of the extracts of natural origin, from different sources with potential for use in the food and pharmaceutical industry.

### 4.5. Antioxidant Activity by Voltammetric Analysis

Under different compounds in extracts of *M. albida* with antioxidant properties being able to produce synergistic effects, the total antioxidant potential (TAP), defined as the measure of the overall antioxidant activity of all antioxidants in a given sample, generally differs from the sum of the antioxidant capacities of the separate compounds [64,65]. This parameter was obtained by the electrochemical technique, cyclic voltammetry (CV). In addition, the technique was used as a diagnostic tool in the identification of the largest possible number of antioxidant compounds present in the aqueous extract of *M. albida*. Figure 7A shows the cyclic voltamperogram of the extract using the supporting electrolyte sodium acetate 0.1 mol L^−1^; where it can see two oxidation waves at potentials of 0.45 V and 0.84 V; which suggests the presence in the extract of at least two types of reducing species or a reducing species that can be oxidized by two stable intermediates. The voltamperogram of extract showed no voltammetric waves towards reduction potentials (between potentials of 0.75 V to +0.5 V), which suggests that the reducing species in extract of *M. albida* leaves can indeed act with antioxidant capacity. On the other hand, at 0.12 V a reduction wave is present that we can consider as response of the process to potential of 0.45 V. If this is the case, it would be a response to the presence of quinone groups formed in the oxidation scans from OH^−^ groups present in structures of antioxidant species.

The latter again suggests that antioxidant species are present in the extract studied. In order to corroborate the results obtained by CV, DPV was used; a technique that allows for the obtaining of current-potential signals with greater sensitivity [66], since it can eliminate the contribution of background current obtained in cyclic voltamperograms, which do not allow for a good definition of voltammetric signals, and can also deconvolve the current signals in order to demonstrate the existence of other additional waves in the system.

In the voltamperogram obtained by DPV (Figure 8B), the presence of two oxidation waves can be observed in the ranges of potentials obtained by CV; results that confirm the existence in the extract of two species with antioxidant capacity. The values of currents obtained for the two-voltammetric waves are in the same order of magnitude, which indicates that both species are present in similar amounts in the extract. On the other hand, if we consider that each of the signals corresponds to a species with chemical characteristics and individual behavior, the species have different antioxidant capacity, one species with greater antioxidant capacity, with oxidation potential at 0.34 V, and the other one with lower capacity, with a potential of 0.79 V.

The reducing capacity of *M. albida* extract was confirmed by the cyclic voltammetry evaluation of the *Ma*AgNPs (Figure 9). Figure 9A shows the voltamperogram of an aqueous solution of AgNO_3_ 5 mmol L^−1^, where a reduction peak is observed at +0.027 V and an oxidation peak at +0.559 V vs. Ag/AgCl. The reduction signal is associated with the electrodeposition of silver ions on the surface of the vitreous carbon electrode, whereas the oxidation is associated with the redissolution of the silver deposited on the electrode in the reduction sweep.

The cyclic voltamperogram obtained from the *Ma*AgNPs (Figure 9B), does not show reduction waves, while an oxidation signal is observed at +0.3792 V. The absence of a cathodic signal suggests the non-existence or limited presence of Ag^+^ ions in the said extract, which shows that it is mostly made up of Ag^0^ nanoparticles, which are responsible for the oxidation signal at the potential of +0.3792 V. This result also demonstrates the ability of the *M. albida* extract to stabilize the Ag^0^ nanoparticles.

## 5. Conclusions

In this research, highly dispersed and stable silver nanoparticles (6.5 ± 3.1 nm) were synthesized using aqueous leaf extract of *Mimosa albida* by the green synthesis route. The reduction of metallic silver ions was associated with a large number of antioxidants present in the *Mimosa albida* extract. Polyphenols such as apigenin and luteolin are probably responsible for the biopotency of the extracts; these compounds are presumably linked to the antioxidant potential observed in the extract, which in turn was useful to produce *Ma*AgNPs. The amount of total polyphenols, as well as the phenolic profile and antioxidant capacity, show changes when comparing the extract with the *Ma*AgNPs, thus suggesting a reducing effect of the polyphenolic compounds present. Since the *Ma*AgNPs obtained showed antioxidant capacity, they could potentially be used in the phytochemical industry.

## Figures and Tables

**Table 1:** Energy-dispersive X-ray spectroscopy (EDX) elemental analysis of *Ma*AgNPs.

Element	C	O	Na	Mg	S	K	Ca	Ag
Norm. wt%	68.22	25.50	3.50	0.44	0.91	0.54	0.44	0.44
σ	7.34	0.76	0.37	0.03	0.04	0.08	0.02	0.08

**Table 2:** Total olyphenol content in *M. albida* and *Ma*AgNPs.

Sample	Total Polyphenol (Mg GAE 100 g^−1^ Dry Mass)
*M. albida* extract	1320.4 ± 17.6
*Ma*AgNPs	257.3 ± 5.1

**Table 3:** Antioxidant capacity of the extract of *M. albida* and *Ma*AgNPs.

Sample	ORAC-FL (µmol TE/g of Extract)
*M. albida* extract	86,917 ± 6287
*Ma*AgNPs	7563 ± 967
